# Methamphetamine Induces Dopamine D1 Receptor-Dependent Endoplasmic Reticulum Stress-Related Molecular Events in the Rat Striatum

**DOI:** 10.1371/journal.pone.0006092

**Published:** 2009-06-30

**Authors:** Subramaniam Jayanthi, Michael T. McCoy, Genevieve Beauvais, Bruce Ladenheim, Kristi Gilmore, William Wood, Kevin Becker, Jean Lud Cadet

**Affiliations:** 1 Molecular Neuropsychiatry Research Branch, National Institute of Drug Abuse, National Institutes of Health (NIH)/Department of Health and Human Services (DHHS), Intramural Research Program, Baltimore, Maryland, United States of America; 2 Gene Expression and Genomics Unit, National Institute of Aging, National Institutes of Health (NIH)/Department of Health and Human Services (DHHS), Intramural Research Program, Baltimore, Maryland, United States of America; James Cook University, Australia

## Abstract

Methamphetamine (METH) is an illicit toxic psychostimulant which is widely abused. Its toxic effects depend on the release of excessive levels of dopamine (DA) that activates striatal DA receptors. Inhibition of DA-mediated neurotransmission by the DA D1 receptor antagonist, SCH23390, protects against METH-induced neuronal apoptosis. The initial purpose of the present study was to investigate, using microarray analyses, the influence of SCH23390 on transcriptional responses in the rat striatum caused by a single METH injection at 2 and 4 hours after drug administration. We identified 545 out of a total of 22,227 genes as METH-responsive. These include genes which are involved in apoptotic pathways, endoplasmic reticulum (ER) stress, and in transcription regulation, among others. Of these, a total of 172 genes showed SCH23390-induced inhibition of METH-mediated changes. Among these SCH23390-responsive genes were several genes that are regulated during ER stress, namely ATF3, HSP27, Hmox1, HSP40, and CHOP/Gadd153. The secondary goal of the study was to investigate the role of DA D1 receptor stimulation on the expression of genes that participate in ER stress-mediated molecular events. We thus used quantitative PCR to confirm changes in the METH-responsive ER genes identified by the microarray analyses. We also measured the expression of these genes and of ATF4, ATF6, BiP/GRP78, and of GADD34 over a more extended time course. SCH23390 attenuated or blocked METH-induced increases in the expression of the majority of these genes. Western blot analysis revealed METH-induced increases in the expression of the antioxidant protein, Hmox1, which lasted for about 24 hours after the METH injection. Additionally, METH caused DA D1 receptor-dependent transit of the Hmox1 regulator protein, Nrf2, from cytosolic into nuclear fractions where the protein exerts its regulatory functions. When taken together, these findings indicate that SCH23390 can provide protection against neuronal apoptosis by inhibiting METH-mediated DA D1 receptor-mediated ER stress in the rat striatum. Our data also suggest that METH-induced toxicity might be a useful model to dissect molecular mechanisms involved in ER stress-dependent events in the rodent brain.

## Introduction

Methamphetamine (METH) abuse is a complex neuropsychiatric disorder that affects a significant number of adolescents and adults worldwide. METH addicts often use large quantities of the drug [1], [2] and can suffer for drug-induced psychosis, withdrawal-associated depression, coma and/or death after taking accidental overdoses [3]. Neuroimaging studies have also revealed a number of abnormalities in the brains of these patients. These include loss of striatal dopamine (DA) transporters and of serotonin transporters [4], [5] and evidence of reactive microgliosis [6]. Previous post-mortem studies are in agreement with some of those results [7]. Moreover, METH can cause degeneration of monoaminergic systems and neuronal apoptosis in various brain regions including the rodent striatum [8]–[10]. METH-induced cell death is dependent, in part, on activation of endoplasmic reticulum (ER)-dependent death pathways [11].

The ER is an intracellular organelle which is involved in diverse arrays of cellular functions. The ER is very densely packed with enzymes that are involved in quality control of protein synthesis and post-translational modification including folding of proteins [12], [13]. Malfunctions in these processes result in misfolded and/or unfolded proteins that can accumulate in the ER, with consequent activation of compensatory reactions such as the unfolded protein response [14]. If these compensatory mechanisms fail to restore cellular homeostasis, cell death ensues via activation of ER-dependent apoptosis [15], [16]. The signaling pathways that mediate compensatory mechanisms and cell death during ER stress are regulated by the ER-resident chaperone, BiP/GRP78 (Binding Immunoglobulin Protein/Glucose Response Protein 78), which is bound to three ER transmembrane sensor proteins, namely, activating transcription factor 6 (ATF6), inositol requiring enzyme 1α (Ire1α), and protein kinase R-like ER kinase (PERK) [17]. During ER stress, BiP dissociates from these proteins and triggers a series of events that lead to oligomerization of IRE1α [18] and of PERK [19] as well as cleavage and translocation of ATF6α into the nucleus [20]. Ire1α has site-specific endoribonuclease (RNase) activity which splices and activates X-box Binding Protein-1 (XBP1) mRNA and leads to increased production of XBP1 protein [18] which regulates the transcription of genes that participate in the maintenance of protein folding and ER-associated degradation (ERAD) [21]. PERK oligomerization leads to phosphorylation of eukaryotic translation initiation factor 2α (eIF2α) and inhibition of global translation [22], but increased activating transcription factor 4 (ATF4) translation [23]. Periods of excessive ER stress also cause ATF4-, ATF6, and XBP1-dependent up-regulation of the pro-apoptotic transcription factor, C/EBP homologous protein (CHOP) [24] which appears to mediate cell death, in part, by causing up-regulation of pro-death members of Bcl-2 family proteins such as BIM [25]. The accumulated evidence supports the involvement of ER stress and related molecular events in neurodegenerative events [26] including METH-induced neuronal apoptosis [11].

The present study was therefore undertaken to document if blockade of DA D1 receptor function might influence METH-induced gene expression because of the evidence that the DA D1 receptor antagonist, SCH23390, can protect against METH toxicity in the rat striatum [9], [27]. Thus, the purpose of this paper is to report that microarray and quantitative PCR analyses detected METH-induced increases in the expression of activating transcription factor 3 (ATF3), heat shock protein 27 (HSP27), heme oxygenase 1 (Hmox1), heat shock protein 40 (HSP40), CHOP, and BIP that are known ER stress-responsive genes. The METH-induced changes were attenuated, to variable degrees, by pretreatment with SCH23390. Western blot analysis also documented changes in Hmox1 and nuclear factor erythroid 2-related factor 2 (Nrf2) proteins which are both involved in cellular protective mechanisms. These results demonstrate that METH-induced neurotoxicity occur, in part, through DA D1-mediated and ER-dependent molecular events.

## Results

### METH- and SCH23390-induced differential gene expression in the rat striatum

**Figure 1:**
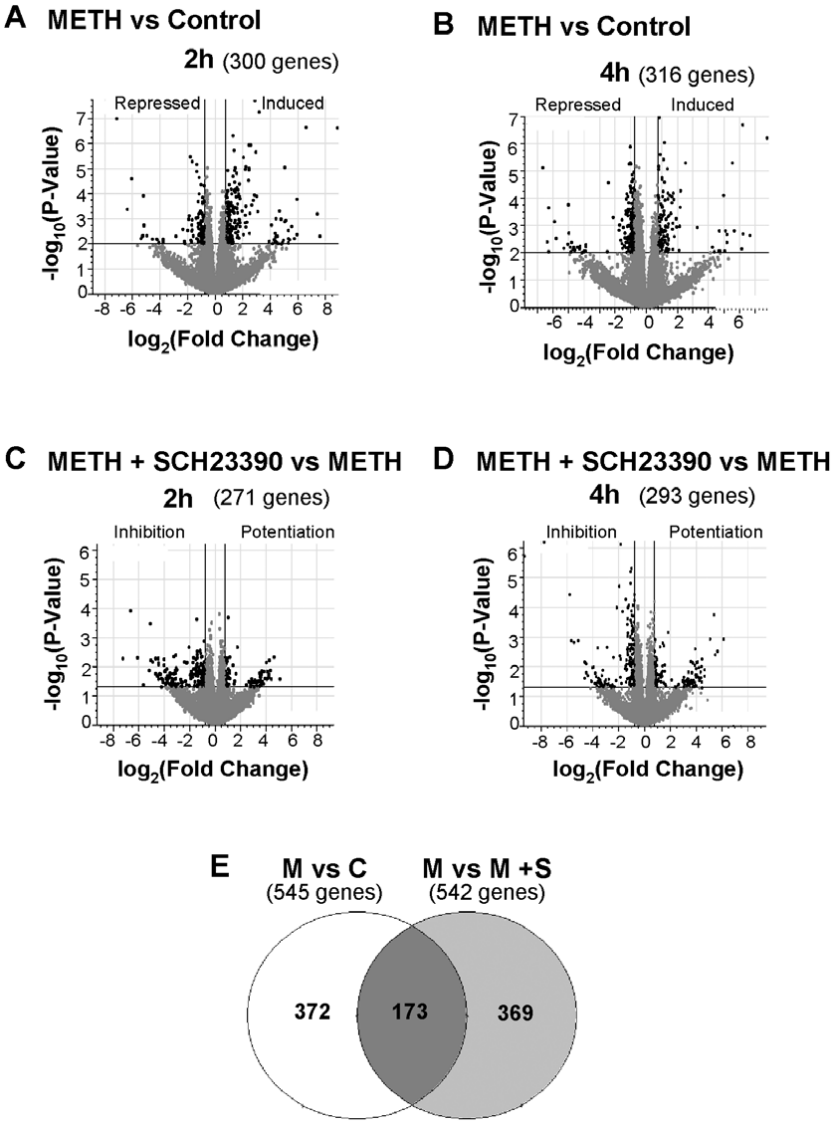
METH- and SCH23390-induced differential gene expression in the rat striatum. (A and B) Volcano plots showing the effects of METH on striatal gene expression. Comparison of METH-treated rats to control rats revealed that METH caused changes in (A) 300 genes at 2 h after the METH injection, of which 189 were induced and 111 were repressed, and (B) 316 genes at the 4 h time-point, of which 133 were induced and 183 were repressed. The Volcano plots showing the effects of SCH23390 pre-treatment on METH-induced striatal gene expression are shown in (C) and (D). These comparisons revealed that these two groups show differences in the expression (C) of 271 genes at the 2 h time-point, the expression of 184 of which was inhibited and of 87 of which was potentiated by SCH23390 (D) of 293 genes at the 4 h time-point, the expression of 175 of which was inhibited and of 118 of which was potentiated by SCH23390. (E) The Venn diagram shows the overlap of genes that are common between the two different comparisons: M vs. C and M vs. M+S. Abbreviations are C, control; S, SCH23390; M, methamphetamine. To be identified as SCH23390-responsive, the genes must have passed the following criteria: greater or less than 1.7-fold changes at p<0.05.The 173 METH-responsive genes that were influenced by pre-treatment with SCH23390 are listed in Table S3 and the SCH23390-resistant genes are listed in Table S4.

To identify METH-regulated genes, RNA samples from striata of rats treated with saline, METH, SCH23390, or combination of these drugs were subjected to microarray analysis using Rat Illumina arrays which allow for query of 22,227 transcripts (see Tables S1 and S2 in Supplemental data for the complete set of array data obtained from animals euthanized at 2 and 4 hours after METH treatment, respectively). Figure 1 shows the effects of SCH23390 on METH-induced gene expression in the rat striatum. The Volcano plots in figures 1A and 1B show that, in comparison to the control group, METH caused changes in the expression of 300 and 316 genes at 2 and 4 hours, respectively, after injection of the drug. Because 71 genes were significantly changed at both time points, this left a combined total of 545 genes that were identified as METH-responsive at these times. Figures 1C and 1D show the Volcano comparisons between the SCH23390 and the METH+SCH23390 groups at 2 and 4 hours, respectively. There were a total of 271 and 293 genes that were differentially expressed at those times. The Venn diagram in Figure 1E reveals that the expression of 173 METH-responsive genes was influenced by pre-treatment with SCH23390. Table S3 in Supplemental data summarizes the fold-changes and the functional classifications for these genes. The SCH23390-resistant genes are listed in Table S4 in Supplemental data. The METH-responsive genes participate in apoptotic pathways, control of cell adhesion, regulation of cell cycle, ER stress, signal transduction, and transcription regulation (see Tables S3 and S4 for more details).

Because METH has previously been shown to cause neuronal apoptosis, in part, by activating the ER-dependent death pathway [11] and because SCH23390 can protect against METH-induced toxicity [9], we decided to focus more attention on the effects of METH and SCH23390 on genes that are involved in responses to ER stress. Table 1 shows a total of 27 ER-related genes that were either induced (23) or downregulated (4) by the METH injection. SCH23390 pretreatment caused inhibition of METH-induced changes in 15 genes; these are listed in bold in the table. These include Armet, ATF3, BiP, CHOP, Dnajb1 (DnaJ (Hsp40) homolog, subfamily B, member 1), Hmox1, HSP27, among others.

**Table 1:** METH administration causes changes in ER stress-related genes.

Accession No.	Gene Symbol	Time			
		2 h	2 h	4 h	4 h
		M/C	(M+S)/M	M/C	(M+S)/M
**NM_012912**	**Atf3**	**166.534**	**−23.355**	**2.325**	**−1.959**
**NM_031970**	**Hspb1 (HSP27)**	**27.016**	**−4.723**	**9.659**	**−7.562**
**NM_012580**	**Hmox1**	**19.956**	**−6.583**	**3.173**	**−2.739**
**XM_341663**	**Dnajb1 p**	**8.458**	**−2.552**	**2.597**	**−2.141**
**NM_022934**	**Dnaja1 (HSP40)**	**2.975**	**−1.659**	**2.262**	**−2.052**
XM_218543	Plekhf1	2.942	−1.116	1.618	−1.307
XM_215722	Dnajb4 p	2.829	−1.448	2.092	−1.504
NM_080906	Ddit4 (REDD1)	2.678	1.142	1.455	−1.195
**NM_024134**	**Ddit3 (CHOP, Gadd153)**	**2.557**	**−1.340**	**2.301**	**−2.483**
**XM_223680**	**Ahsa2 p**	**2.543**	**−1.083**	**2.997**	**−2.488**
**NM_053612**	**Hspb8 (HSP22)**	**2.378**	**−1.671**	**1.889**	**−1.712**
**NM_012935**	**Cryab**	**2.295**	**1.331**	**1.806**	**−1.716**
**XM_233767**	**Dnajb5 p**	**2.003**	**−1.715**	**2.016**	**−2.049**
NM_175761	Hspca (HSP90)	1.908	−1.380	1.604	−1.585
XM_217147	Dnaja4	1.862	−1.490	1.647	−1.651
NM_022522	Casp2	1.722	−1.481	1.226	−1.292
**NM_031122**	**St13**	**1.550**	**−1.083**	**1.767**	**−1.773**
**NM_013083**	**Hspa5 (BIP, GRP78)**	**1.314**	**1.304**	**2.563**	**−2.042**
**NM_053523**	**Herpud1 (Herp)**	**1.304**	**1.332**	**2.055**	**−1.931**
**XM_236614**	**Armet p**	**1.061**	**1.396**	**2.063**	**−1.882**
NM_022232	Dnajc3	1.185	1.085	1.726	−1.650
NM_001004210	Xbp1	1.154	1.207	1.723	−1.608
**NM_053849**	**Pdia4**	**−1.141**	**1.460**	**1.781**	**−1.765**
XM_576401	Phldb1	−1.200	−1.222	−1.944	1.226
NM_152790	Carhsp1	−1.217	−1.014	−1.833	1.337
XM_234909	Plekhj1	−1.430	−1.040	−2.060	1.695
XM_230610	Hspa12b p	−3.243	2.005	−1.233	1.155

The data in this table were generated from the comparisons between the METH and control groups at 2 and 4 h according to the criteria used in the analyses (greater or less than 1.7-fold and p<0.01). The values represent fold changes between the specified groups (n = 6). The genes are listed in descending order according to the METH-induced fold changes at the 2 h time point. The bolded genes are genes whose METH-induced expression was significantly inhibited by the SCH23390 pretreatment.

### SCH23390 attenuates METH-induced activation of ATF6-regulated gene expression

**Figure 2:**
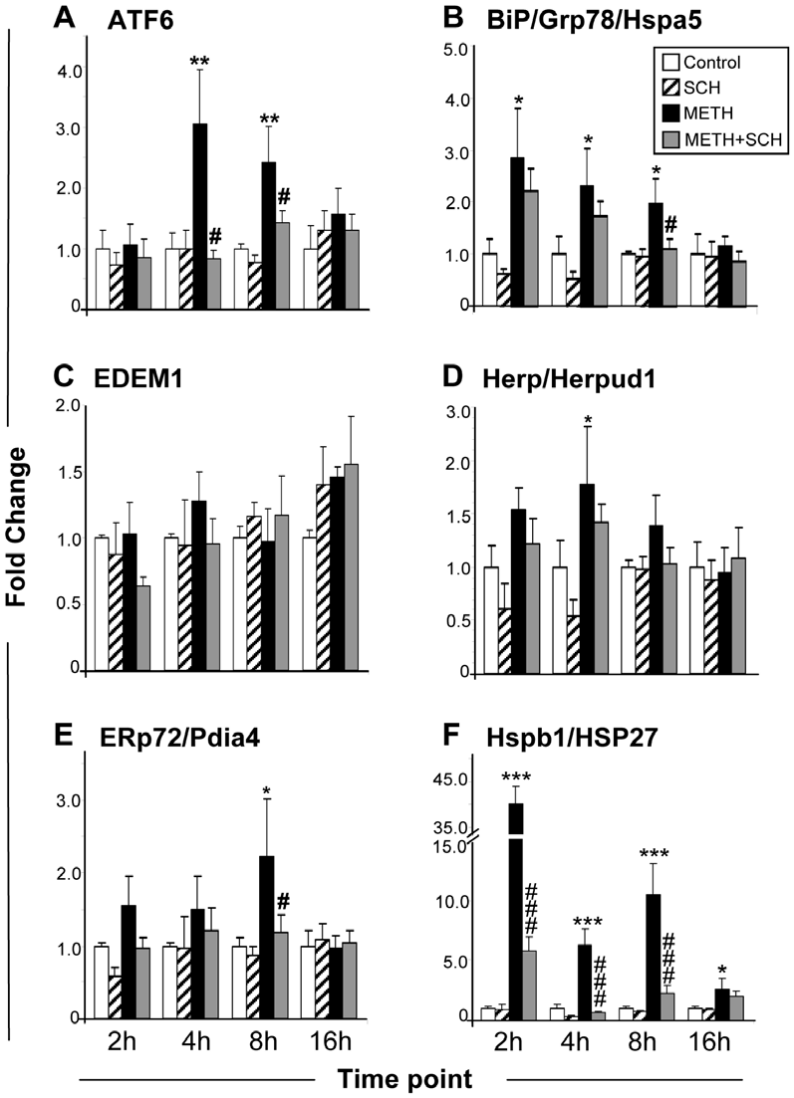
Effects of METH and SCH23390 on the expression of ATF6 and some ATF6-regulated genes. METH administration caused early induction of (A) ATF6 (B) BiP and (F) Hspb1/HSP27 transcripts. The METH-induced changes in the expression of these genes were normalized by pre-treatment of SCH-23390. (D, E) Herp/Herpud1 and ERp72/Pdia4 showed only transient changes at 4 h and 8 h respectively, after METH treatment whereas (C) EDEM1 was not affected. Data were obtained from RNA isolated from six animals per group and determined individually. The levels of mRNA were normalized to 18 s rRNA levels. Values represent means±SEM of fold changes relative to the controls. Statistical significance was determined by ANOVA followed by protected least-squares difference (PLSD). Key to statistics: * = M vs. C; # = M vs. M+S; *, # p<0.05; **, ## p<0.01; ***, ### p<0.001.

ER stress is associated with unfolded protein response activation which is associated with the production of protective chaperones and antioxidant enzymes [28]. However, excessive ER stress can cause cellular demise via the activation of the ER-dependent apoptotic pathway [16], [24]. The activation of these protective and pro-apoptotic genes is regulated by BiP interaction with ATF6, IRE1α, and PERK [14], [28]. ER stress interferes with these interactions and leads to the activation of ER signaling pathways [14], [28]. Figure 2 shows the effects of METH on some genes that are involved in the ATF6-dependent pathway [29]. ATF6 mRNA was induced by METH in a DA D1 receptor-dependent fashion at both 4 and 8 hours after drug administration (Fig. 2A). Consistent with the array data, METH caused significant increases in BiP mRNA, with the peak changes being observed at 2 hours and normalization by 16 hours (Fig. 2B). SCH23390 was only able to suppress the effects of METH at 8 hours post-drug. This is consistent with the array data which did not identify BiP as a SCH23390-sensitive gene at 2 and 4 hours. HSP27 mRNA also showed marked biphasic DA D1 receptor-mediated increases after the METH injection, reaching ∼40-fold at 2 hours after METH, decreasing to 5-fold at 4 hours and then reaching another smaller peak at 8 hours after METH (Fig. 2F). Other genes that we tested, namely ER degradation enhancer, mannosidase alpha-like 1 (EDEM1) (Fig. 2C), homocysteine-inducible, endoplasmic reticulum stress-inducible, ubiquitin-like domain member 1 (Herpud1) (Fig. 2D), and endoplasmic reticulum protein 72 (ERp72) (Fig. 2E), show transient or no changes after the METH injection. Specifically, there were no significant changes in EDEM1 expression in any of the treatment groups at any of the times studied. There were, however, significant increases in Herpud1 mRNA in the striatum of animals sacrificed at 4 hours after the METH injection; these increases were not significantly attenuated by the SCH23390 pretreatment (Fig. 2D). In addition, the METH injection caused DA D1 receptor-dependent increases in ERp72/Pdia4 expression measured only at 8 hours after injection of the psychostimulant.

### Effects of METH and SCH23390 on XBP1-dependent gene expression

**Figure 3:**
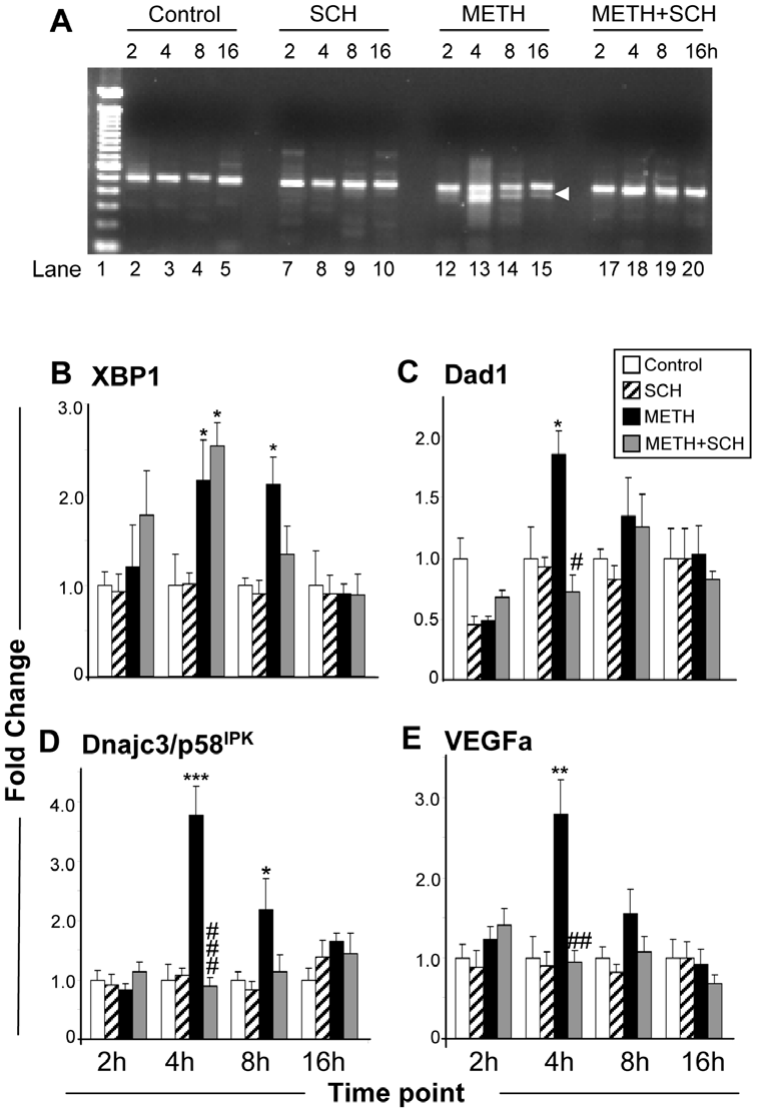
Effects of METH and of SCH23390 on XBP1 target genes. (A) Administration of METH caused splicing of XBP1 mRNA. XBP1 mRNA fragments were amplified by RT-PCR and PCR fragments were separated on 2% agarose gel. Arrowhead indicates spliced XBP1 (479 bp). The sequences of primers used to amplify the XBP1 transcripts are provided in Supplemental Table 1. (B) Levels of unspliced XBP1 mRNA were induced by METH at 4 and 8 h after the drug injection. The induced XBP1 mRNA was not significantly inhibited by SCH23390 pretreatment. METH administration also caused increases in the expression of the XBP1 target genes: (C) DAD1, (D) p58^IPK^, and (E) VEGFa. The METH-induced changes in these three transcripts were attenuated by pretreatment with SCH23390. Statistical analyses and key to statistics are as described in Figure 2.

The transcriptional activity of XBP1 is regulated by IRE1α-dependent splicing of XBP1 mRNA and generation of active XBP1 protein [18], [30]. This is followed by increases in XBP1-dependent increases in the expression of ERAD-related mRNAs [21], [31]. In order to determine if the drug causes activation of IRE1α signaling, we measured XBP1 mRNA splicing at different times after the METH injection. Figures 3A and 3B show that the METH injection resulted in increases in XBP1 splicing and XBP1 transcription at various times after the drug injection. The METH-induced XBP1 mRNA splicing was inhibited by SCH23390 pretreatment. However, METH-induced increases in unspliced XBP1 mRNA were not significantly attenuated by the SCH23390 pretreatment, suggesting that other METH-mediated events, such as oxidative stress [1], might be responsible for these changes. Quantification of three XBP1 target genes revealed that METH administration is associated with increases in the anti-death gene, defender against cell death 1 (Dad1) (Fig. 3C) [32] which is a subunit of the mammalian oligosaccharyltransferase [33]–[35]. The METH-induced changes were attenuated by pretreatment with SCH23390. The expression of DnaJ (Hsp40) homolog, subfamily C, member 3 (Dnajc3/p58^IPK^), a member of the Hsp40 family of chaperones [36]–[38] which can control PERK eIF2α kinase activity during ER stress [39] and protect the stressed ER [40], was also induced by METH in a DA D1 receptor-sensitive fashion (Fig. 3D). There were also significant DA D1 receptor-mediated increases in the expression of vascular endothelial growth factor (VEGF) (Fig. 3E) which is a neuroprotective factor [41], [42] that protects against ER stress-dependent neurodegeneration [43].

### METH-mediated induction of genes of the ER PERK-dependent pathway

**Figure 4:**
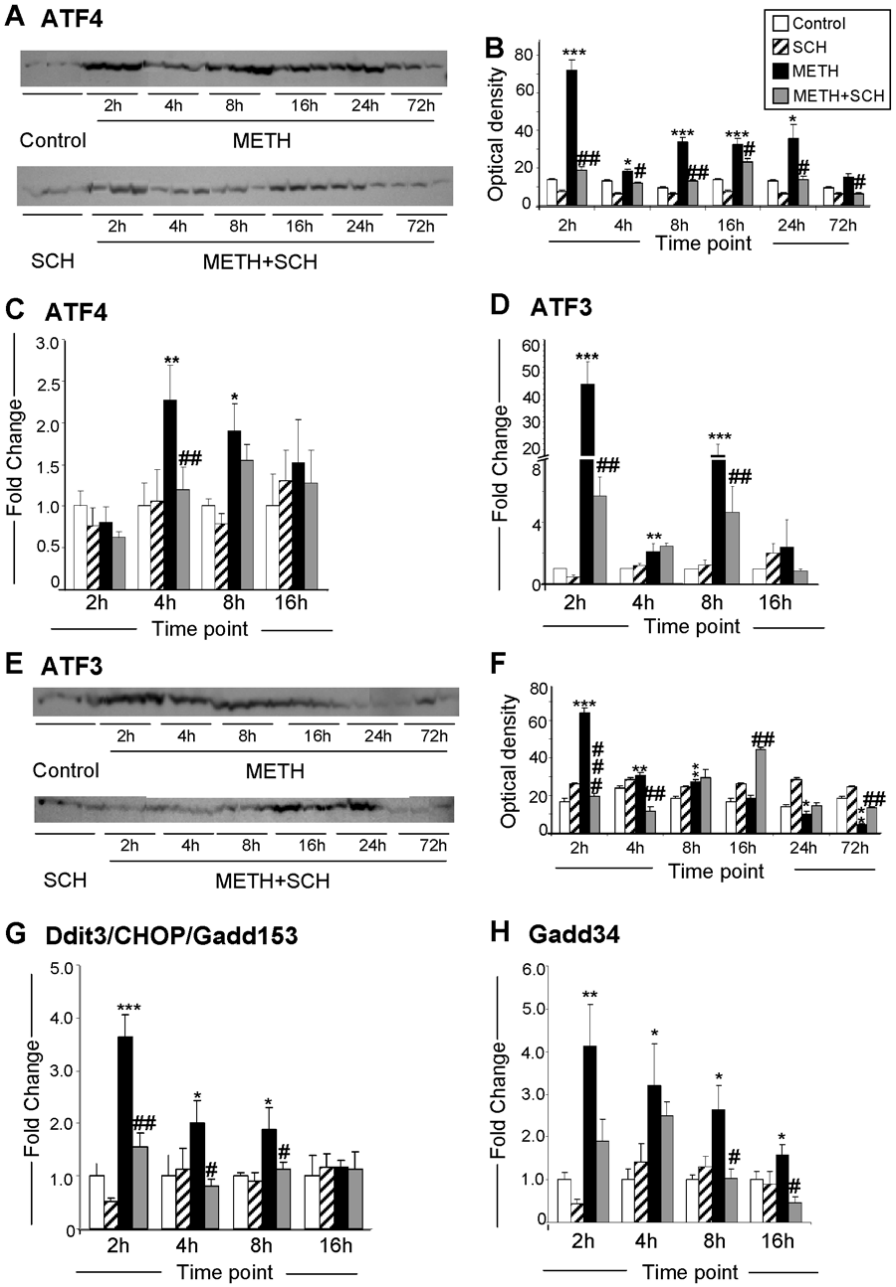
SCH23390 pretreatment causes inhibition of METH-induced changes in ER PERK-dependent gene expression. (A, B) METH caused significant DA D1 receptor-dependent increases in ATF4 protein. (C) METH-induced changes in the levels of ATF4 mRNA and their inhibition by SCH23390. (D) Effects of METH caused on ATF3 mRNA; (E, F) ATF3 protein expression; (G, H) Effect of METH on the levels of CHOP/Gadd153 and Gadd34 mRNA. Statistical analyses and key to statistics are as described in Figure 2.

As mentioned above, ER stress is also associated with activation of the PERK-dependent pathway [22], [23], [44]. Figure 4 shows the effects of METH and of SCH23390 on the expression of 4 genes that participate in the PERK-regulated pathway [45]. As shown in Figs 4A and 4B, METH caused significant DA D1 receptor-dependent increases in ATF4 protein which occurred as early as 2 hours after the METH injection. These results are consistent with the fact that ER stress is known to induce early increases in ATF4 translation [46]. Injection of SCH23390 significantly prevented the METH-induced increases in ATF4 protein levels (Fig. 4B). There were also METH-induced increases in ATF4 mRNA which occurred later than the changes in ATF4 protein, with SCH23390 inhibiting these changes (Fig. 4C). As stated above, ATF4 is a member of the ATF/CREB family of transcription factors which is upregulated during ER stress [46] and which controls the expression of ATF3 and CHOP [47], [48]. Consistent with the array, we observed that METH caused substantial increases in ATF3 mRNA (Fig. 4D). These increases were biphasic, reaching ∼45-fold at 2 hours, down to 3-fold at 4 hours, and back up to 18-fold at 8 hours after the METH injection. Expression of ATF3 protein was also induced by METH in a DA D1 receptor-dependent fashion (Figs. 4E and 4F). The levels of CHOP/Gadd153 mRNA also showed significant DA D1 receptor-sensitive METH-mediated increases (Fig. 4G). The increases in CHOP expression are consistent with previous results obtained in the striata of METH-treated mice [11]. CHOP is known to participate in stress-induced apoptosis and to regulate the expression of several genes including growth arrest and DNA-damage-inducible 34 (GADD34) [49]. As observed in Fig. 4H, the levels of GADD34 mRNA, a protein that dephosphorylates eIF2α [50], also showed substantial METH-induced increases that are suppressed by SCH23390 in a time-dependent fashion.

**Figure 5:**
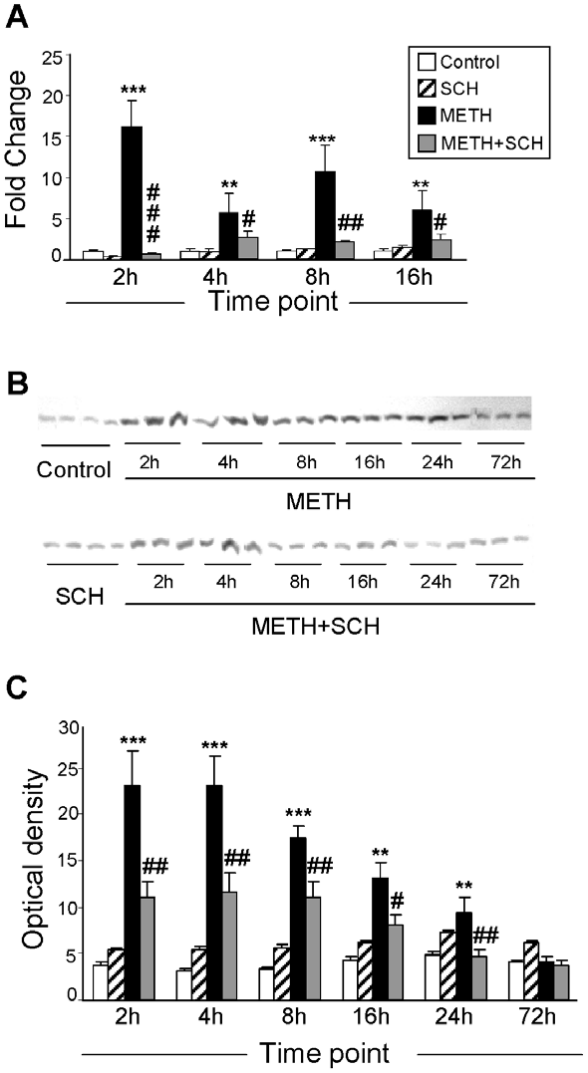
Pre-treatment of SCH-23390 inhibits METH-induced changes in Hmox1 expression. (A) METH caused biphasic changes in Hmox1 mRNA levels in rat striatum. (B, C) Western analyses also showed significant increases in the levels of Hmox1 protein. Pre-treatment of SCH-23390 normalized Hmox1 expression in a time-dependent manner. Representative photomicrographs show results of three samples per time point of METH and METH+SCH – treated rats and four samples per time point of control and SCH-treated rats are shown in (B). Abbreviations are as described in Figure 1. (C) The quantitative data of western blot data represent means±SEM (n = 6). For quantification, the signal intensity was normalized over the signal intensity of tubulin. Statistical analyses and key to statistics are as described in Figure 2.

Figure 5 shows the effects of METH on Hmox1 which is an important antioxidant enzyme which can be induced by a number of pharmacological agents [51], [52]. As per the microarray data, METH caused significant SCH23390-sensitive increases in Hmox1 mRNA which remained elevated up to 16 hours after the drug injection (Fig. 5A). METH also caused marked DA D1 receptor-sensitive increases in Hmox1 protein expression which lasted for about 24 hours (Figs. 5B and 5C).

**Figure 6:**
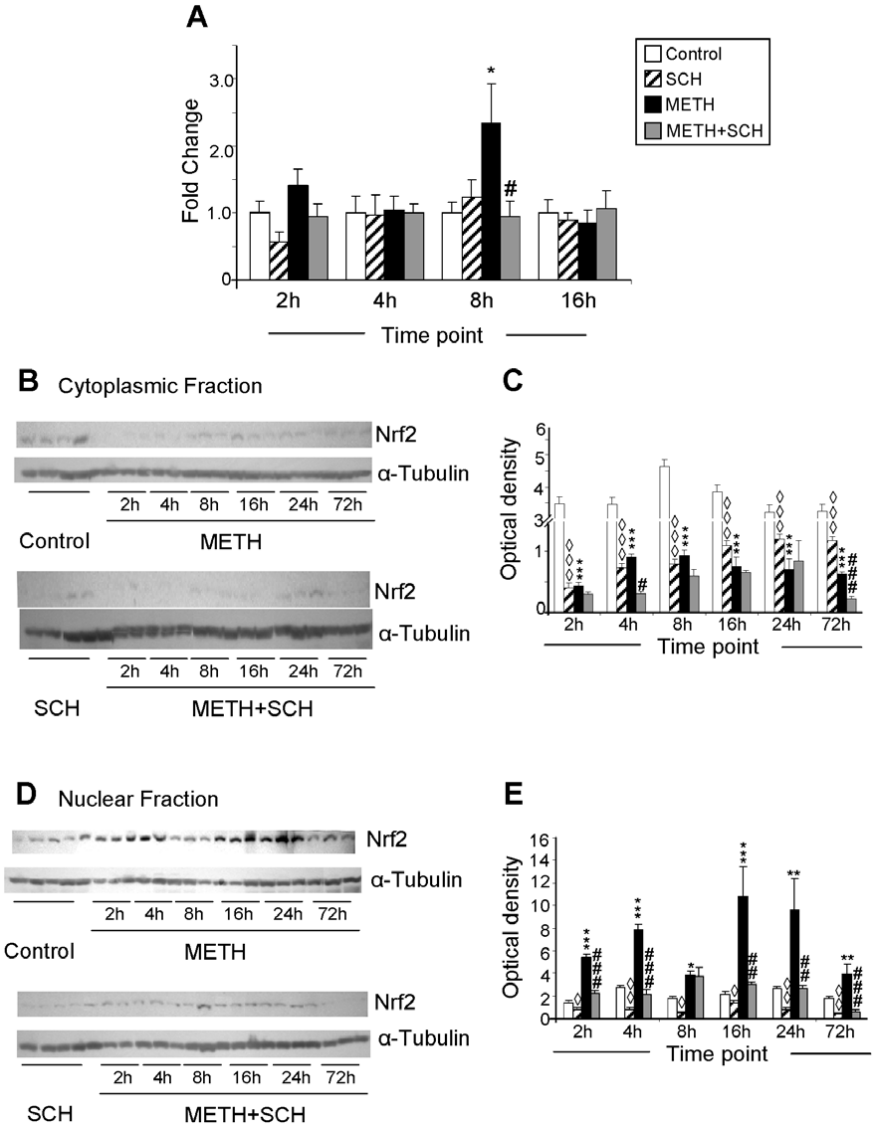
METH treatment causes transit of Nrf2 from the cytoplasm to the nucleus in the rat striatum. (A) Effects of METH and SCH23390 Nrf2 mRNA. METH administration causes the shuttling of Nrf2 protein from the cytosol (B) to the nucleus (D). The METH-induced increases in nuclear Nrf2 protein was inhibited by SCH23390 pre-treatment. Nuclear and cytosolic fractions were separated as described in the Materials and methods section. The fractions were obtained from individual samples of six animals per time-point. Representative photomicrographs show results of three samples per time point of METH and METH+SCH− treated rats and four samples per time point of control and SCH-treated rats. The membranes were reprobed with α-tubulin antibody to confirm equal protein loading. Signal intensity was measured densitometrically with LabWorks version 4.5 (BioImaging Systems analysis software, BioImaging System, UVP Inc., Upland, CA) and the quantitative data (C, E) are represented as optical density of six animals per time-point (means±SEM). For quantification, the signal intensity was normalized over the signal intensity of tubulin. Statistical significance was determined by ANOVA followed by protected least-squares difference (PLSD). Key to statistics: * = M vs. C; ^∧^ = S vs. C; # = M vs. M+S. *, ◊, # p<0.05; **, ◊◊, ## p<0.01; ***, ◊◊◊, ### p<0.001.

Because Hmox1 is regulated, during ER stress, via PERK-mediated Nrf2 phosphorylation and its transit from the cytosol into the nucleus [53]–[55], we also tested the possibility that METH might cause changes in Nrf2 mRNA and protein levels. Figure 6A shows that METH caused only transient DA D1 receptor-dependent increases in Nrf2 mRNA levels. In contrast, the METH injection was associated with decreases in the levels of Nrf2 protein in the striatal cytosolic fractions and its prolonged accumulation in the nuclear fractions (Figs. 6B–6E). The quantitative data in Fig. 6C show that the levels of cytosolic Nrf2 protein in the control group of animals remained relatively constant while there were significant decreases in cytosolic Nrf2 levels in the SCH23390 and METH-treated rats. The changes in METH-induced accumulation of Nrf2 protein in nuclear fractions occurred in a biphasic fashion, with initial changes observed as early as 2 hours after the drug but with some reversal towards normalization by 8 hours. This was followed by another phase of increases at 16 hours lasting up to 72 hours after the injection of METH (Fig. 6E). The METH-induced increases in nuclear Nrf2 were significantly inhibited by SCH23390. However, there were further decreases in cytosolic Nrf2 levels in the rats treated with both METH and SCH23390, decreases that were significantly different from those observed in the METH group (Fig. 6C). These changes are not due to increased transit into the nucleus since SCH3390 blocked the effects of METH on Nrf2 accumulation in the nucleus (compare Figs. 6C and 6D). These observations are consistent with the observation that SCH23390 caused decreases in cytosolic Nrf2 (Fig. 6C) without causing increases in nuclear Nrf2 (Fig. 6E).

## Discussion

The ER is an essential organelle which is responsible for post-translational processing and proper folding of recently synthesized proteins that participate in secretory pathways and are membrane bound [12], [13]. The ER can be stressed by dysfunctions in calcium homeostasis, oxidative stress, and improper protein folding [14], [16]. These abnormalities trigger ER stress-dependent events which consist of the production of chaperone proteins that attempt to prevent cellular demise [14] and/or activation of cell death cascades when ER stress is too overwhelming [16], [56]. In the present study, we have found that the illicit neurotoxin, METH, can cause the activation of ER stress-mediated gene expression. Our microarray analyses identified several genes whose transcript levels were significantly increased early after injection of the drug. METH administration also caused significant activation of genes and proteins that are downstream of the ATF6, IRE1alpha, and PERK ER signaling pathways [14], [16]. These include both protective and pro-death transcripts. Quantitative PCR confirmed these changes and also provided more detailed time courses for these changes. We also found that METH-mediated activation of ER stress-dependent events is dependent, in part, on the stimulation of DA D1 receptors that are very abundant in the rat striatum [57]. These observations are consistent with reports that stimulation of D1 receptors by DA can cause death of neuroblastoma cells [58] and of striatal neurons [59] in cultures.

Our findings that METH can cause ER stress in the rat striatum are consistent with our prior observations that METH can cause apoptosis of mouse striatal cells via cross-talks between ER- and mitochondria-dependent death pathways [11]. Although we have previously shown METH-induced increases in CHOP and BiP expression in the mouse striatum [11], the present data provide a more detailed picture of METH-responsive ER stress genes in the rat striatum. As previously reported in the mouse striatum, METH administration caused significant increases in the transcription of CHOP which is known to be involved in pathways that lead to neuronal apoptosis [60]. The present results also extend our previous observations by showing that the increases in CHOP expression are dependent on stimulation of striatal DA D1 receptors. The METH-induced increases in CHOP expression are also preceded by significant increases in the levels of ATF4 protein, a member of the ATF/CREB class of transcription factors, which is a regulator of CHOP expression during ER stress [48]. During ER stress, activation of the PERK-dependent pathway leads to phosphorylation of eIF2α [22], [23], [61]. Phosphorylation of this protein leads to global inhibition of translation, which results in the reduction of ER protein load. In addition, eIF2α phosphorylation leads to increased translation of a number of mRNAs [62], one of which is ATF4 [23], [44], [47]. Our observations of METH-induced increases in the expression of ATF4 protein and transcript are consistent with prior reports that this transcription factor is induced under conditions of ER and oxidative stresses [44], [63]. The findings that METH caused increases in ATF4 protein earlier than the changes in ATF4 mRNA levels are consistent with ER stress-induced increased translation of ATF4 [23], [47], [50]. During ER stress, ATF4 also regulates the expression of ATF3 [48] which is also increased after METH administration in a DA D1 receptor-dependent fashion. Of note, ATF3 is induced by an array of cellular stresses [64] and by administration of the METH analog, amphetamine [65]. Interestingly, ATF3 is also thought to be an important player in the integral stress response (ISR) [23]. It needs to be mentioned that adenoviral over-expression of ATF3 in PC12 cells prevents c-Jun-N-Terminal kinase (JNK)-dependent apoptosis via increased expression of the chaperone, HSP27 [66]. This report is consistent with our demonstration that HSP27 is significantly induced by METH. When taken together, those observations suggest that activation of the PERK-ATF4-ATF3 ER stress cascade might be, in part, responsible for adaptive responses of striatal cells to METH-induced oxidative stress and for counterbalancing the pro-apoptotic effects of CHOP induction by the drug.

Our results also suggest that METH treatment can activate IRE1α-dependent transduction mechanisms since the drug induces DA D1 receptor-mediated XBP1 mRNA splicing, which is mediated by IRE1α –associated RNase activity [18]. Several targets have been identified for this transcription factor, among which are Dad1, Dnajc3/p58^IPK^, and VEGF, all of which show DA D1 receptor-dependent METH-induced upregulation (see Fig. 3). The METH-induced increases in their expression suggest that the injection of toxic doses of the drug is associated with the triggering of protective mechanisms in an attempt to prevent METH-induced neuronal apoptosis. This statement is supported by the evidence that Dad1 is an inhibitor of apoptosis [67], whose absence is associated with developmental abnormalities in Dad-1 null mice [68]. In addition, p58^IPK^, first identified as a 58-kDa protein that inhibits PKR in influenza virus-infected kidney cells [37], can complex with PKR and PERK and inhibit their autophosphorylation [39] as well as protect the stressed ER [40]. Thus, the present observation of METH-induced Dnajc3/p58^IPK^ does suggest its participation in a coordinated response to attenuate or prevent METH-mediated deleterious effects on the ER in conjunction with VEGF which has been shown to protect neurons against ischemia [41], [69] glutamate toxicity [70] and ER stress [43]. VEGF appears to exert its protective effects against neuronal apoptosis, in part, by both caspase-dependent [71] and caspase-independent [72] mechanisms. Although similar mechanisms have been identified in METH-induced neuronal apoptosis [73], the potential role of VEGF against METH toxicity has yet to be investigated.

Mammalian HSPs, which include HSP27, HSP40, and HSP70, are molecular chaperones that participate in the proper folding of proteins and help to maintain their native conformations during stressful events [74]–[76]. HSPs also participate in the transfer of improperly folded proteins to the proteasome for degradation. HSPs are induced by heat shock, hypoxic and ischemic events, and oxidative stress [74], [75]. Recent studies have documented a role for these proteins in neurodegenerative processes and have demonstrated that HSPs are important in cellular protection against aggregation-prone proteins and in animal models of neurodegeneration [77], [78]. Thus, our demonstration of METH-induced expression of the chaperones, Hsp27/Hspb1 [79] and BiP/Grp78 [80], suggests that striatal cells are able to mount adaptive defensive HSP-modulated networks against the toxic effects of the drug. This statement is consistent with the fact that a BiP inducer, called BiP inducer X, was able to reduce the number of apoptotic cells in the penumbra of ischemic strokes caused by occlusion of the middle carotid artery of the mouse [81]. That contention is further supported by reports that BiP can protect against ER stress-induced cell death in several models of cellular demise [82], [83]. In addition to the HSP70 chaperones, HSP27 has been shown to be a very potent neuroprotective agent. The chaperone protects against cell death caused by alpha-synuclein *in vitro* [84], as well as against kainate toxicity [85] and cerebral ischemia [86] *in vivo*. HSP27 appears to exert its protective effects, in part, by inhibiting caspase-dependent apoptotic pathways [87]–[89]. Because increased expression of HSP27 is observed in cells that survive ischemic insults [90], it is not far-fetched to suggest that the induction of these chaperones might occur in cells destined to survive against METH-induced apoptosis. This assumption will need to be demonstrated in future studies.

In addition to the HSPs, METH administration also caused significant increases in the expression of Hmox1 which is a phase 2 enzyme that is induced by oxidative stress and cellular injury [91], [92]. Hmox1 catalyzes the rate-limiting step in heme degradation and is involved in protecting cells against various pathological insults including ischemic injuries and peroxide toxicity [93], [94]. We found that the METH injection caused sustained increases in the levels of Hmox1 mRNA and protein in a DA D1 receptor-regulated fashion. The observations that METH causes oxidative stress [95] and that METH-induced toxicity can be prevented by blocking the DA D1 receptor with SCH23390 [9] suggest that METH might induce Hmox1 transcription via two mechanisms, one mediated by generation of oxygen-based radicals and another occurring via receptor stimulation since DA can cause cell death by stimulation of DA D1 receptor [59], [96]. Our findings that METH can also cause shuttling of Nrf2 protein from cytosolic to nuclear fractions are thus consistent with the published literature indicating that the regulation of Hmox1 transcription involves Nrf2 protein phosphorylation and its stabilization and transit from the cytosol and accumulation into the nucleus [53], [54], [97], [98]. It is also of interest that the combined administration of the DA D1 antagonist, SCH23390, with METH, led to further reduction of cytosolic Nrf2 levels without concomitant increases in nuclear Nrf2. In fact, the pretreatment with SCH23390 blocked the METH-induced nuclear accumulation of Nrf2 (see Fig. 6). Also of note is the fact that SCH23390, given alone, also caused reduction of cytosolic Nrf2 without increasing nuclear Nrf2 (compare Figs. 6C and 6E). When taken together, these observations suggest that blockade of DA neurotransmission via D1 receptors might cause increases in proteasomal degradation of Nrf2 [99]. Furthermore, the consistency of the data on SCH23390-induced effects at all the time points examined suggests potentially important roles for DA D1 receptors in the regulation of ubiquitin-dependent proteasomal degradation in the mammalian basal ganglia [100]. The veracity of this idea will need to be tested experimentally.

Because the transcription of both Hmox1 and NQO1 is dependent, in part, on Nrf2 [101]–[103], it is of interest to discuss the induction of Hmox1 by METH in relation to the protection afforded by NAD(P)H quinone oxidoreductase-1(NQO1) induction against METH toxicity in vitro [104]. Miyazaki and collaborators [104] reported that BHA, a known NQO1 inducer [105], was able to increase NQO1 and to provide significant protection against METH toxicity in vitro. Because the authors also found that METH, itself, also caused NQO1 induction [104], their findings and our present observations indicate that toxic doses of the psychostimulant activate a protective cascade which is mediated via Nrf2-regulated transcriptional events, which involve coordinated induction of several genes that code for phase II detoxification enzymes including Hmox1 and NQO1 [101].

As noted above, the ER is involved in the processing and folding of membrane and secretory proteins [12], [13]. Depletion of ER calcium, oxidative stress, and disturbances in protein folding, which all cause ER stress, are accompanied by increases in the expression of ER chaperones[14], [106] including ERp72 [107]. ERp72/Pdia4 is a member of the family of protein disulfide isomerase [108] which participates in the formation and reduction of disulfide bonds and their isomerization [109]. Our observation of METH-induced increases in ERp72 expression is consistent with the reported up-regulation of ERp72 during ER stress [110]. Similar to our results, it has been reported that ERp72 and Hmox1 are both up-regulated after depletion of ER calcium stores [111].

In summary, the present study demonstrates that a toxic dose of METH can cause changes in the transcription of genes that are involved in the regulation of an array of cellular functions. These include activation of genes that participate in cellular responses to ER and oxidative stresses. The present observations are the first to document that stimulation of striatal DA D1 receptors can lead to substantial activation of ER stress-dependent gene expression in that brain structure. These results also implicate ER signaling pathways as important players in METH-induced long-term neurobiological effects. Finally, our results suggest that the METH toxicity model might be useful in investigations seeking to dissect the molecular control of the ER stress-related molecular events via stimulation of G-protein coupled receptors in the mammalian brain.

## Materials and Methods

### Animals and Drug Treatment

All animal use procedures were according to the NIH Guide for the Care and Use of Laboratory Animals and were approved by the NIDA (National Institute of Drug Abuse) Animal Care Committee. Male Sprague Dawley rats (Charles River Labs), weighing 250–300 g, were used. Rats were injected with either saline or METH (40 mg/kg) via the intraperitoneal route in the presence or absence of the DA D1 receptor antagonist, SCH23390 (1 mg/kg). The dose of METH was chosen because it can cause long-term depletion of monoaminergic terminals [112] and neuronal apoptosis [113], [114] in the rodent brain. The dose of SCH23390 was chosen because similar doses protect against METH toxicity [9]. This approach resulted in four groups of animals: Saline control (C), saline plus SCH23390 (1 mg/kg) (S), saline plus METH (40 mg/kg) (M), and SCH23390 followed by METH (M+S). SCH23390 was given 30 minutes before the single injection of saline or METH. For the microarray analysis, animals (*n* = 6 per group) were decapitated and striatal tissues were harvested at 2 and 4 hours after the METH or control injections.

### RNA extraction

Total RNA was isolated using Qiagen RNeasy Midi kit (Qiagen, Valencia, CA) according to the manufacturer's instructions. RNA integrity was assessed using an Agilent 2100 Bioanalyzer (Agilent, Palo Alto, CA).

### Microarray hybridization and scanning

Microarray hybridization was carried out using Illumina's RatRef-12 Expression BeadChips arrays (22, 227 probes) (Illumina Inc., San Diego, CA), In brief, a 600 ng aliquot of total RNA from each striatal sample was amplified using Ambion's Illumina RNA Amplification kit (cat. no. IL1791; Ambion, Austin, TX). Single-stranded RNA (cRNA) was generated and labeled by incorporating biotin-16-UTP (Roche Diagnostics GmbH, Mannheim, Germany, cat. no. 11388908910). 750 ng of each cRNA sample were hybridized to Illumina arrays at 55°C overnight according to the Illumina Whole-Genome Gene Expression Protocol for BeadStation (Illumina Inc., San Diego, CA, cat. # 11201828). Hybridized biotinylated cRNA was detected with Cyanine3-streptavidine (Amersham Biosciences, Piscataway, NJ, cat. #146065) and quantified using Illumina's BeadStation 500GX Genetic Analysis Systems scanner.

### Microarray data analysis

All microarray data reported in the manuscript is described in accordance with MIAME guidelines. The complete raw data for the analyses done at 2 and 4 hours after the METH injection are listed in supplement tables S1 and S2, respectively. The Illumina BeadStudio software was used to measure fluorescent hybridization signals. Data were extracted by BeadStudio (Illumina, San Diego, CA) and then analyzed using GeneSpring software v. 7.3.1 (Silicon Genetics, Redwood City, CA, USA). Raw data were imported into GeneSpring and normalized using global normalization. The normalized data were used to identify genes as METH-responsive if they show increased or decreased expression according to the arbitrary cut-off of 1.7-fold changes at p<0.01. The genes that were identified as SCH23390-responsive showed an increase or decrease expression according to the arbitrary cut-off of 1.7-fold changes at p<0.05.

### Quantitative RT-PCR Analysis

Total RNA was extracted from striatal samples from all the 4 groups and was used for quantitative RT-PCR to confirm the results obtained with microarray. Quantitative RT-PCR were carried out with minor modification of previously described procedures [9]. Briefly, individual total RNA (1 µg) samples from six rats for each time-point for every group were reverse-transcribed into cDNA using Advantage RT for PCR kit (Clontech, Mountain View, CA, USA). PCR experiments employed a primer set and iQ SYBR Green Supermix (BioRad, Hercules, CA USA) using the Chromo4 RT-PCR Detection System (BioRad). Sequences for gene-specific primers corresponding to PCR targets were obtained using LightCycler probe design software v. 2.0 (Roche, Indianapolis, IN, USA) and were synthesized at the Synthesis and Sequencing Facility of Johns Hopkins University (Baltimore, MD). The primer sequences are listed in Table S5 in the supplement. The relative amounts of messenger RNA (mRNA) normalized to 18 s rRNA were then quantified.

### PCR analysis of XBP1 splicing

cDNA synthesis from total RNA is similar to the procedure described under the section quantitative RT-PCR analysis. To amplify XBP1 mRNA (NM_005080), PCR was performed for 35 cycles (95°C for 30 s; 58°C for 30 s; 72°C for 1 min) using the PCR primers 5′-AGA GTA GCA GCT CAG ACT GCC AG -3′ and 5′-PCGA ACT GGG TCC TTC TGG GTA-3′ and with iQ SYBR Green Supermix (BioRad, Hercules, CA USA) using the Chromo4 RT-PCR Detection System (BioRad). Fragments representing spliced and unspliced XBP1 were visualized on 2% agarose gels with ethidium bromide staining.

### Immunoblot Analysis

Striatal tissues were first washed with chilled 0.01 M PBS. Cytoplasmic and nuclear protein fractions were prepared using NE-PER nuclear and cytoplasmic extraction reagents based on the manufacturer's instruction (Pierce, Rockford, IL). Protein concentration of cell lysates was determined with the BioRad Dc Protein assay reagent (BioRad, Temecula, CA). The lysates were then denatured with sample buffer (62.5 mM Tris-HCl, 10% glycerol, 2% SDS, 0.1% bromophenol blue, and 50 mM DTT) at 100°C for 5 min, and subjected to SDS-PAGE. Proteins were electrophoretically transferred to Hybond-PTM membrane (Amersham Pharmacia Biotech, Piscataway, NJ) and incubated overnight at 4°C with ATF3, ATF4 (1∶500 dilution), Nrf2 and Hmox1 antibodies (1∶750 dilution, Santa Cruz Biotechnology Inc., Santa Cruz, CA, USA). After washing in Tris-buffered saline with 0.1% Tween-20, membranes were pre-incubated in the detergent/blocking buffer containing 1∶2000 horseradish peroxidase (HRP)-conjugated anti-rabbit secondary antibody (Cell Signaling Technology Inc., Danvers, MA, USA) for 1 hr at room temperature. To confirm equal protein loading, blots were re-probed with α-tubulin antibody (1∶4000; Sigma, 2 h at RT). LumiGLO chemiluminescent reagents (Cell Signaling Technology Inc., Danvers, MA, USA) were used to detect protein expression. Signal intensity was measured densitometrically with LabWorks version 4.5 (BioImaging Systems analysis software, BioImaging System, UVP Inc., Upland, CA). For quantification, the signal intensity was normalized over the signal intensity of tubulin.

### Statistical Analysis

Statistical analysis for qRT-PCR and Western blot data was performed with the statistical package StatView (SAS Institute, Cary, NC, USA) using ANOVA followed by Fisher's protected least-significant difference test (p<0.05).

## Supporting Information

Table S1 Raw data of all genes at 2 h after METH or control injections.
(20.68 MB XLS)

Table S2 Raw data of all genes at 4 h after METH or control injections.
(14.57 MB XLS)

Table S3 List of 173 SCH23390-sensitive METH-responsive genes in the rat striatum.
(0.07 MB XLS)

Table S4 List of 372 SCH23390-resistant METH-responsive genes in the rat striatum.
(0.12 MB XLS)

Table S5 List of primer sequences used for quantitative RT-PCR.
(0.02 MB XLS)
